# The three dimensions of caregiver grief in dementia caregiving: Validity and utility of the subscales of the Marwit‐Meuser Caregiver Grief Inventory

**DOI:** 10.1002/gps.5238

**Published:** 2019-12-06

**Authors:** Ivana Chan, Philip Yap, Shiou Liang Wee, Tau Ming Liew

**Affiliations:** ^1^ Geriatric Education and Research Institute Singapore; ^2^ Department of Geriatric Medicine, Khoo Teck Puat Hospital Singapore; ^3^ Health and Social Sciences Cluster Singapore Institute of Technology Singapore; ^4^ Department of Geriatric Psychiatry Institute of Mental Health Singapore; ^5^ Psychotherapy Service Institute of Mental Health Singapore; ^6^ Saw Swee Hock School of Public Health National University of Singapore Singapore

**Keywords:** caregiver grief, dementia, Marwit‐Meuser Caregiver Grief Inventory, predeath grief, reliability, validity

## Abstract

**Background:**

The experience of grief in family caregivers as they provide care for persons with dementia is often overlooked. The Marwit‐Meuser Caregiver Grief Inventory (MM‐CGI) is one among the few scales that capture such experiences. In a recent study, MM‐CGI was found to contain three subscales identifying dimensions of loss in caregivers—*Personal‐Sacrifice Burden* (*PSB*), *Heartfelt Sadness, Longing, and Worry* (*HSLW*), and *Felt Isolation* (*FI*). We aimed to evaluate the validity and utility of these dimensions in a multiethnic Asian population.

**Methods:**

Family caregivers (n = 394) completed MM‐CGI and scales assessing caregiver burden, depression, and gains. Internal consistency reliability was examined using Cronbach α; test‐retest reliability using intraclass correlation coefficient; and construct validity using Pearson correlation coefficient. The utility of the MM‐CGI dimensions was evaluated by comparing caregivers with high subscale scores across dementia stages and caregiving relationship.

**Results:**

The three dimensions of MM‐CGI exhibited adequate internal consistency, test‐retest reliability, construct validity, and known‐group validity. *PSB* correlated most strongly with caregiver burden (*r* = 0.78); *HSLW* with caregiver depression (*r* = 0.75); and *FI* with caregiver burden and caregiver depression (*r* = 0.60, respectively). Caregivers with high total grief scores tended to experience most difficulty with *HSLW* (90.8%), followed by *PSB* (75.4%) and *FI* (46.2%). The three dimensions also increased across the dementia stages, with *FI* higher in mild dementia, *PSB* higher in moderate dementia, and *HSLW* higher in severe dementia. Spousal caregivers experienced most difficulty in *HSLW*, whereas children caregivers experienced similar levels of difficulty across the dimensions.

**Conclusions:**

The three dimensions of MM‐CGI captured distinct aspects of caregiver grief in a multiethnic Asian population and would enable more individualized assessments and interventions for caregiver grief.

**Key Points**

- The three domains of MM‐CGI possessed adequate psychometric properties to capture distinct aspects of caregiver grief.
- The three domains provided more specific information on the grief profile experienced by caregivers, across the different stages of dementia and caregiving relationship.
- The three domains demonstrated added utility in bringing attention to individuals who may possibly require assistance for specific aspects of caregiver grief but may otherwise be overlooked as they did not meet the cut‐off score based on the total score of MM‐CGI.

## 1 INTRODUCTION

By the year 2030, there will be over 65 million persons with dementia (PWDs) worldwide, with the numbers estimated to almost double every 20 years.^1^ Due to individual preferences, limited vacancies, and high costs of residential care facilities, a large proportion of PWDs is expected to live at home and cared for by their family members. Hence, corresponding to the growth in the number of PWDs is the rapid increase in the number of family caregivers. Dementia caregivers have been found to have a higher risk of developing physical and mental health conditions,^2^ and caregivers who experience higher levels of loss and grief before the death of the PWD are at risk of health problems after the PWD passes on.^3^ Research also indicates that the magnitude of grief that caregivers experience preceding the death of the PWD has been considered to be greater than what is experienced after the death of the PWD.^4^, ^5^, ^6^ These vulnerabilities and risks that caregivers experience necessitate effective interventions for them, especially with regard to their experience of loss and grief preceding the death of the PWD.^7^, ^8^

Although there are services tailored to assist caregivers such as caregiver training programs, respite care, and day care, most of the interventions commonly focus on reducing caregiver burden and improving coping skills.^9^ These interventions may not have sufficiently addressed the feelings of sadness and guilt that caregivers experience before the physical death of the PWD. Indeed, a recent systematic review highlighted the unmet emotional needs of dementia caregivers, describing their experiences as “feeling bereft.”^10^ Other terms used to describe the concept of caregiver grief include “anticipatory grief,”^4^ “ambiguous loss,”^11^ and “pre‐death grief.”^12^

While there has yet to be a consensus regarding the definition of caregiver grief, it typically encompasses emotive responses as caregivers mourn for the psychological and physical changes in PWDs and anticipate impending losses long before actual death occurs.^12^ Other important precepts surrounding the experience of caregiver grief comprise factors unique to caring for PWDs, such as communication challenges, asynchronous loss, and an ambiguous disease trajectory leading to worry and uncertainty about the future.^12^ Moreover, findings suggest that caregiver grief is intermittently present, with caregivers experiencing a waxing and waning of emotions such as sorrow and yearning throughout the trajectory of dementia, from diagnosis to end of life.^5^, ^13^ Precursors to caregiver grief include an emotional attachment between the caregiver and PWD and the caregiver's experience of perceived losses with regard to personal freedom, relationships, or the PWD's identity.^12^ Given the array of emotions experienced throughout the course of caregiving, caregivers undergoing caregiver grief have been shown to suffer from negative outcomes such as depression,^14^ caregiver burden,^15^ and a desire to institutionalize the PWD prematurely.^16^ In a recent longitudinal study, caregiver grief was reported to predict caregiver depression 2.5 years later, even after accounting for the effects of caregiver burden.^14^ Caregiver grief was further shown to act in synergy with burden, whereby high levels of grief amplified the effect of burden on caregiver depression at baseline.

The negative effects of high levels of grief warrant a need for a validated instrument that can identify individuals with caregiver grief for targeted support. The MM‐CGI, along with its abbreviated versions (eg, 18‐item MM‐CGI‐Short Form [MM‐CGI‐SF],^17^ 6‐item MM‐CGI‐Brief Form,^18^ or 4‐item Screening Tool for Caregiver Grief^19^), is one of the few validated measures of caregiver grief. The initial validation study in the United States identified three main factors in the MM‐CGI: (1) *Personal Sacrifice Burden* (*PSB*); *(2) Heartfelt Sadness and Longing* (*HSL*); and (3) *Worry and Felt Isolation* (*WFI*). *PSB* expresses individual losses due to the caregiving role, such as loss of personal freedom, compromised health, and loss of sleep, in the caregiver's life. *HSL* focuses on the caregiver's intrapersonal emotional reactions to actual or impending loss and appears to be more closely linked to the traditional understanding of grief. *WFI* captures the feelings of worrying about future losses and losing both the support from, and connections with, others. However, in a recent validation study within an Asian multiethnic population,^20^, ^21^ the original factor structure of MM‐CGI was found to fit poorly. Further investigation using exploratory factor analysis revealed that items relating to the “Worry” component in *WFI* loaded in *HSL* instead. Thus, the authors proposed the following revised factor structure for MM‐CGI: (a) *Personal Sacrifice Burden* (*PSB*); (b) *Heartfelt Sadness, Longing and Worry* (*HSLW*); and (c) *Felt Isolation* (*FI*). The current study examined the psychometric properties of the newly proposed factors in the same multiethnic Asian population. Specifically, we evaluated whether the subscales of MM‐CGI (*PSB*, *HSLW*, and *FI*) were reliable and valid in capturing the different dimensions of caregiver grief in dementia caregiving. In addition, we also assessed whether these subscales provided any added future utility in capturing caregiver grief, beyond what is detectable by the total scores of MM‐CGI. We hypothesized that the use of the three dimensions may provide more specific information on the grief profile experienced by caregivers. Furthermore, the use of the domains may help identify grief in specific subdomains, which may otherwise be overlooked if total scores were below the proposed threshold. To that end, the use of the three dimensions may have additional utility of contributing to individualized caregiver grief assessment and targeted support.

## 2 METHODS

### 2.1 Participants and procedures

We recruited caregivers through consecutive sampling as they accompanied the PWD to the dementia services of the only two tertiary hospitals serving the North‐Eastern population of Singapore (Institute of Mental Health and Khoo Teck Puat Hospital, KTPH). Caregivers of PWDs were recruited from either the outpatient memory clinic or inpatient geriatric psychiatry wards. The PWDs were being seen for memory difficulties and possible medical problems resulting in changes in function and self‐care. The inclusion criteria of our recruitment were (a) spouse or child of PWD; (b) caring for PWD who is residing in the community; and (c) age ≥ 21 years. Participants completed on‐site a set of self‐administered questionnaires comprising the Marwit‐Meuser Caregiver Grief Inventory (MM‐CGI),^22^ a caregiver burden scale (Zarit Burden Interview, ZBI),^23^ a depression scale (Centre for Epidemiologic Studies Depression Scale, CES‐D),^24^ and information related to the dyad. Participants from one of the recruitment sites (KTPH) also completed an additional scale assessing caregiving gains (Gain in Alzheimer Care Instrument, GAIN).^25^ All participants were asked to mail back a second set of questionnaires 1 week after for the purpose of assessing test‐retest reliability, of which 60% of the participants did. This study received ethical approval from the Domain Specific Review Board of National Healthcare Group, Singapore. The data that support the findings of this study are available from the corresponding author upon reasonable request.

### 2.2 Measures

MM‐CGI is a caregiver grief scale that was empirically developed to capture the various aspects of losses experienced by caregivers.^22^ It comprises 50 items each assessed using a 5‐point Likert scale based on how much caregivers agree with the statements (1 = strongly disagree; 5 = strongly agree) and summed to generate a total score ranging from 50 to 250. According to the initial validation paper, obtaining a total score of >175 classifies as high grief, and high subscale scores were defined as above mean subscale score +1 SD for each of the dimensions.^22^ MM‐CGI has previously been validated in our local population.^14^, ^20^, ^21^, ^26^, ^27^

ZBI is a 22‐item scale that assesses the perceived burden experienced by caregivers of older persons.^23^ The items are rated on 5‐point Likert scales and summed to generate a total score^28^ ranging from 0 to 88. ZBI was shown to contain five subscales—*Burden in the Relationship*, *Emotional Well‐being*, *Social and Family Life*, *Finances*, and *Loss of Control*.^29^ CES‐D is a 20‐item, self‐administered scale which measures depressive symptomatology in the past 1 week.^24^ Each item is scored on a 4‐point Likert scale to reflect the frequency of each depressive symptom, with the total score ranging from 0 to 60. CES‐D has four subscales—*Depressed Affect*, *Somatic Symptoms*, *Interpersonal Problems*, and *Positive Affect*.^24^, ^30^ Gain in Alzheimer Care Instrument (GAIN) measures caregiving gains in dementia. It is assessed on a 5‐point Likert scale, forming a 10‐item scale with a score range of 0 to 40. Higher scores denote higher gains. ZBI,^31^, ^32^ CES‐D,^33^ and GAIN^25^ have previously been validated in the local context.

The stage of dementia was captured using a brief measure based on the descriptors of the three dementia severities described in the revised third edition of Diagnostic and Statistical Manual of Mental Disorders (DSM‐III‐R).^34^ From the three options, participants chose the description that best described the PWD—still capable of independent living (mild stage), needs some assistance with daily living (moderate stage), or needs round‐the‐clock supervision (severe stage). This brief measure was shown to have adequate agreement with the Clinical Dementia Rating Scale (kappa 0.56‐0.6).^35^, ^36^, ^37^ It is also consistent with the dementia severity descriptions reintroduced in DSM‐5.^38^ The presence of severe behavioral problems was indirectly measured through the need for admission to the geriatric psychiatry ward, indicating behavioral problems that were too severe to be managed in the community setting.

### 2.3 Statistical analyses

We evaluated the psychometric properties of the three subscales of MM‐CGI with respect to internal consistency reliability, test‐retest reliability, construct validity, and known‐group validity.

We assessed internal consistency reliability using Cronbach α and test‐retest reliability using intraclass correlation coefficient (ICC), with values ≥0.70 indicating the minimally acceptable reliability.^39^, ^40^ We also examined the item‐rest correlation for each item in the subscales of MM‐CGI (ie, the correlation between an item and the other items within the same subscale) and the Cronbach α of the subscale if that item was removed. The consistency of responses of each item with the rest of the items within the same subscale is indicated by a minimally acceptable item‐rest correlation of ≥0.40. The Cronbach α of a subscale should decrease if an item was removed from the subscale, indicating the importance of the item in maintaining internal consistency reliability.

We assessed construct validity using Pearson correlation with ZBI, CES‐D, and GAIN scales. Correlation coefficient values of >0.50 are regarded as strong, whereas indices of ≤0.50 are regarded as moderate or weak.^41^ We made the following hypotheses regarding construct validity:

1. Among the three MM‐CGI dimensions, *PSB* would correlate most strongly with ZBI. Items in *PSB* focus on personal caregiver‐related losses such as loss of freedom and energy. They are comparable to the construct of caregiver burden captured by ZBI, where items focus on caregiver‐related losses in the domains such as well‐being and control over one's life.^42^
2. Among the three MM‐CGI dimensions, *HSLW* would correlate most strongly with CES‐D. This is due to the overlapping symptoms between grief and depression.^16^, ^43^
3. All three subscales would correlate less strongly with the Finances subscale of ZBI. The Finances subscale (eg, Item 15: “Do you feel that you don't have enough money to take care of your relative in addition to the rest of your expenses?”) is not expected to correspond to the experience of caregiver grief.
4. All three subscales would correlate less strongly with the Interpersonal Problems subscale of CES‐D. The Interpersonal Problems subscale measures one's perception of others' critical reactions. While it is possible for caregivers to experience these feelings, these correlates may not be justifiably associated with caregivers experiencing grief.
5. All three subscales would correlate less strongly, as well as exhibit negative relationships, with the Positive Affect subscale of CES‐D. The Positive Affect subscale measures positive feelings, which is distinct from, as well as opposite to, the feelings of grief as measured by the MM‐CGI subscales.
6. All three subscales would correlate less strongly, as well as exhibit negative relationships, with GAIN. GAIN measures positive outcomes in caregiving, which is a disparate concept from, as well as opposite to, the feelings of caregiver grief.

We assessed known‐group validity by comparing mean scores of the three MM‐CGI subscales between groups known to differ in levels of caregiver grief (caregiving relationship and stage of dementia). Existing literature suggests that spousal caregivers and more severe stage of dementia have been associated with higher levels of caregiver grief.^4^, ^26^, ^44^ Spousal caregivers may experience more grief related to the loss of emotional attachment with the PWD, because they tend to have longer and more intimate bonds with PWDs.^44^ Similarly, the association between the stage of dementia and caregiver grief could be due to the increase in anticipation of losing the PWD to death and the more distinct psychological death of the PWD, as the illness progresses.

Lastly, we examined the utility of the three MM‐CGI subscales by comparing those with high subscale scores (above mean + 1 SD) across the stages of dementia and caregiving relationship. All analyses were performed with Stata (version 14).

## 3 RESULTS

We recruited 394 participants, with a response rate of 87.8%. Table 1 presents the demographic information of participants. The mean age of participants was 53.0 years (SD 10.7). A majority were Chinese (86.6%), children caregivers (86.3%), and primary caregivers (70.8%). Participants reported a mean ZBI score of 34.8 (SD 16.8) and mean MM‐CGI score of 141.4 (SD 33.8). Specifically for each domain of MM‐CGI, participants attained mean scores of 51.5 (SD 13.2) for *PSB*, 74.8 (SD 19.3) for *HSLW*, and 15.2 (SD 4.0) for *FI*.

**Table 1:** Demographic characteristics of caregivers and persons with dementia (n = 394)

Variable	n (%)
Age, mean (SD)	53.0 (10.7)
Female gender, n (%)	236 (59.9)
Ethnicity, n (%)	
Chinese	341 (86.6)
Malay	25 (6.3)
Indian/Eurasian/Others	28 (7.1)
Marital status, n (%)	
Married	271 (68.8)
Single/widowed/divorced/separated	123 (31.2)
Employment status, n (%)	
Not working	123 (31.2)
Working part‐time	52 (13.2)
Working full‐time	219 (55.6)
Educational attainment, n (%)	
Tertiary	125 (31.7)
Secondary or below	269 (68.3)
Relationship with PWD, n (%)	
Child	340 (86.3)
Spouse	54 (13.7)
Coresidence with PWD, n (%)	264 (67.0)
Duration of caregiving in years, mean (SD)	6.8 (6.7)
Primary caregiving role, n (%)	279 (70.8)

Variables related to PWD	
Age, mean (SD)	79.5 (8.2)
Female gender, n (%)	278 (70.6)
Duration of dementia diagnosis in years, mean (SD)	4.5 (3.5)
Diagnosis of dementia before 65 years old, n (%)	48 (12.2)
Stage of dementia, n (%)	
Mild to moderate	225 (57.1)
Severe	169 (42.9)
Behavioral problems, n (%)	22 (5.6)

Abbreviations: SD, standard deviation; PWD, person with dementia.

The three dimensions of loss, as measured by MM‐CGI, exhibited adequate internal consistency (Cronbach α = .95 for *PSB*, .96 for *HSLW*, and.78 for *FI*). Notably, *FI* had relatively lower internal consistency among the three dimensions, although it was still above the minimally acceptable reliability indices of ≥0.70. The specific MM‐CGI items within each factor and their corresponding item‐rest correlation indices are presented in Table S1. The three dimensions also achieved adequate test‐retest reliability (ICC = .87 for *PSB*, .87 for *HSLW*, and.81 for *FI*).

**Figure 1:**
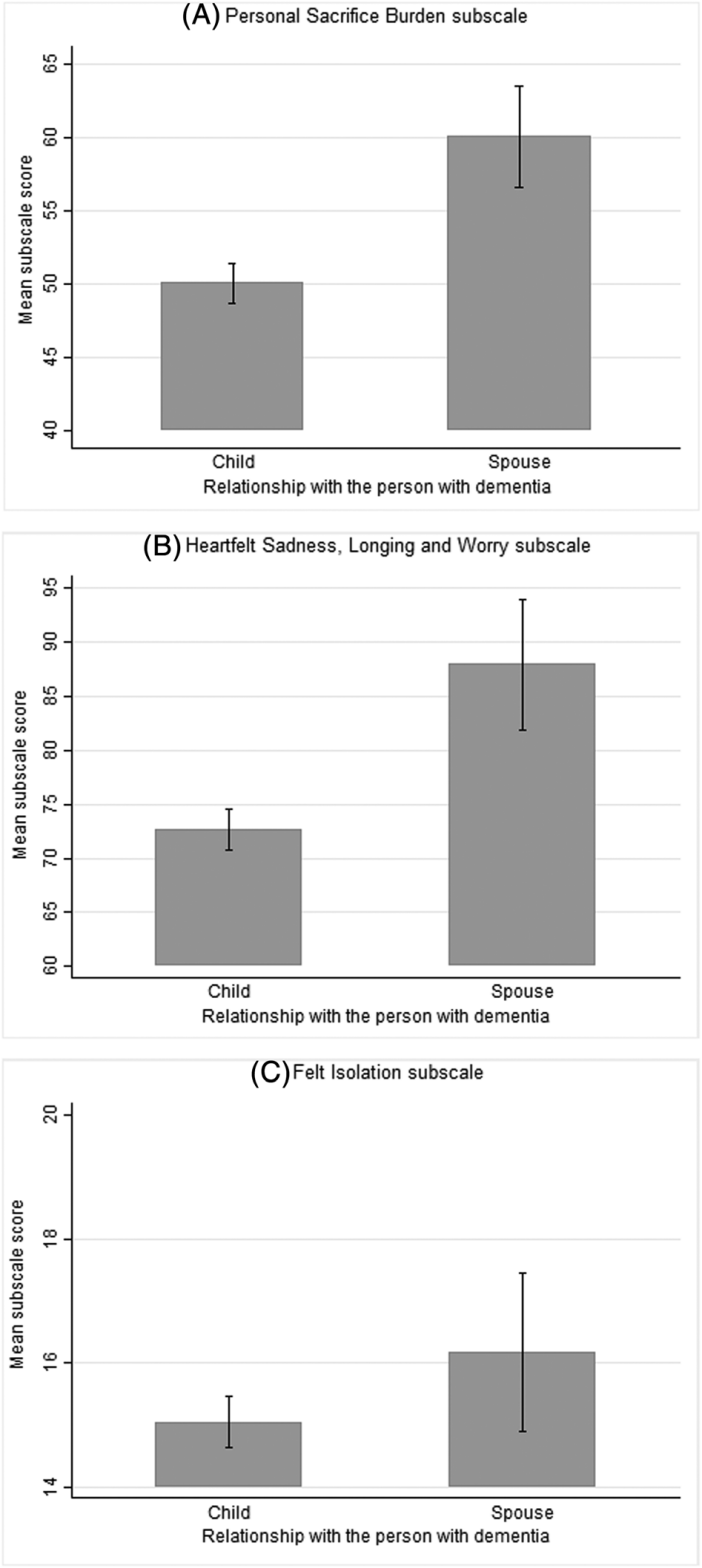
Graph depicting the comparison of mean scores of MM‐CGI, for children and spousal caregivers. MM‐CGI, Marwit‐Meuser Caregiver Grief Inventory

**Figure 2:**
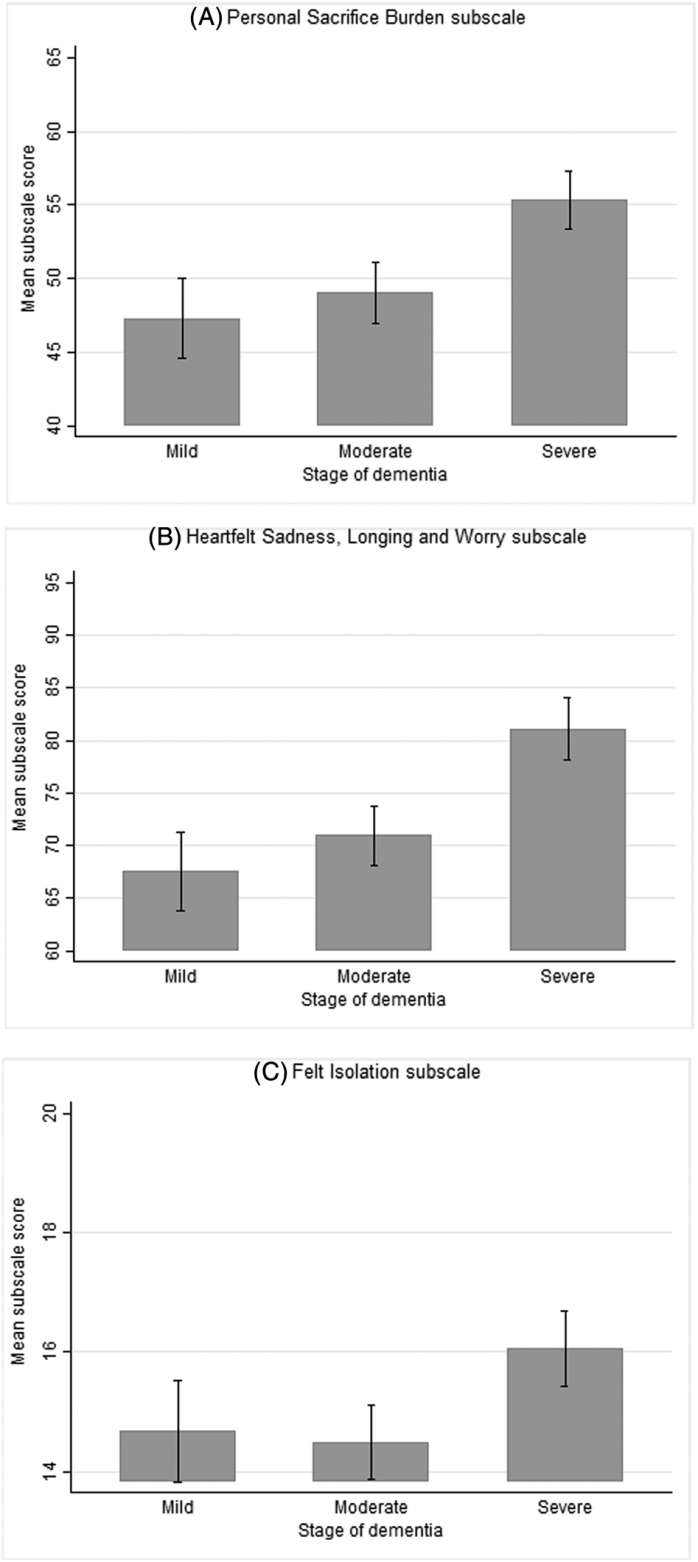
Graph depicting the comparison of mean scores of MM‐CGI, at various stages of dementia. Marwit‐Meuser Caregiver Grief Inventory

The results on construct validity and known‐group validity were consistent with what was hypothesized. The results on construct validity are presented in Table 2. As initially hypothesized, *PSB* correlated most strongly with ZBI (*r* = 0.78, *P* < .001), and *HSLW* correlated most strongly with CES‐D (*r* = 0.75, *P* < .001). Furthermore, as hypothesized, all three MM‐CGI subscales correlated less strongly with the *Finances* subscale of ZBI (0.41 to 0.45), *Interpersonal Problems* (0.40 to 0.49), and *Positive Affect* subscales of CES‐D (−0.40 to −0.32), and GAIN (−0.28 to −0.08). All three MM‐CGI subscales also exhibited negative relationships with the *Positive Affect* subscale of CES‐D and GAIN. The results on known‐group validity are presented in Figures 1 and 2. As observed in Figure 1, spousal caregivers obtained higher mean MM‐CGI scores than children caregivers across all three subscales. Similarly, as observed in Figure 2, mean MM‐CGI scores of caregivers differed at various stages of dementia across all three subscales, with mean scores increasing across the stages of dementia (ie, mild to severe).

**Table 2:** Construct validity as shown by the correlation among various scales, using Pearson correlation coefficient* (n = 394)

	*PSB*	*HSLW*	*FI*
ZBI total scale	0.78	0.68	0.60
*Burden in the Relationship* subscale	0.74	0.61	0.52
*Emotional Well‐being* subscale	0.74	0.64	0.53
*Social and Family Life* subscale	0.72	0.59	0.54
*Loss of Control* subscale	0.66	0.65	0.57
*Finances* subscale	0.45	0.43	0.41
CES‐D total scale	0.69	0.75	0.60
*Depressed Affect* subscale	0.65	0.72	0.52
*Somatic Symptoms* subscale	0.70	0.72	0.56
*Interpersonal Problems* subscale	0.40	0.43	0.49
*Positive Affect* subscale	−0.32	−0.40	−0.38
GAIN scale	−0.08	−0.13	−0.28

Abbreviations: MM‐CGI, Marwit‐Meuser Caregiver Grief Inventory; ZBI, Zarit Burden Interview; CES‐D, Centre for Epidemiologic Studies Depression Scale; *PSB*, *Personal Sacrifice Burden*; *HSLW*, *Heartfelt Sadness*, *Longing*, *and Worry*; *FI*, *Felt Isolation*.

* *P* values (after Bonferroni correction) are <.001 for all estimates, except for GAIN (*PSB*, *P* = 1.00; *HSLW*, *P* = 1.00; *FI*, *P* = .017).

**Figure 3:**
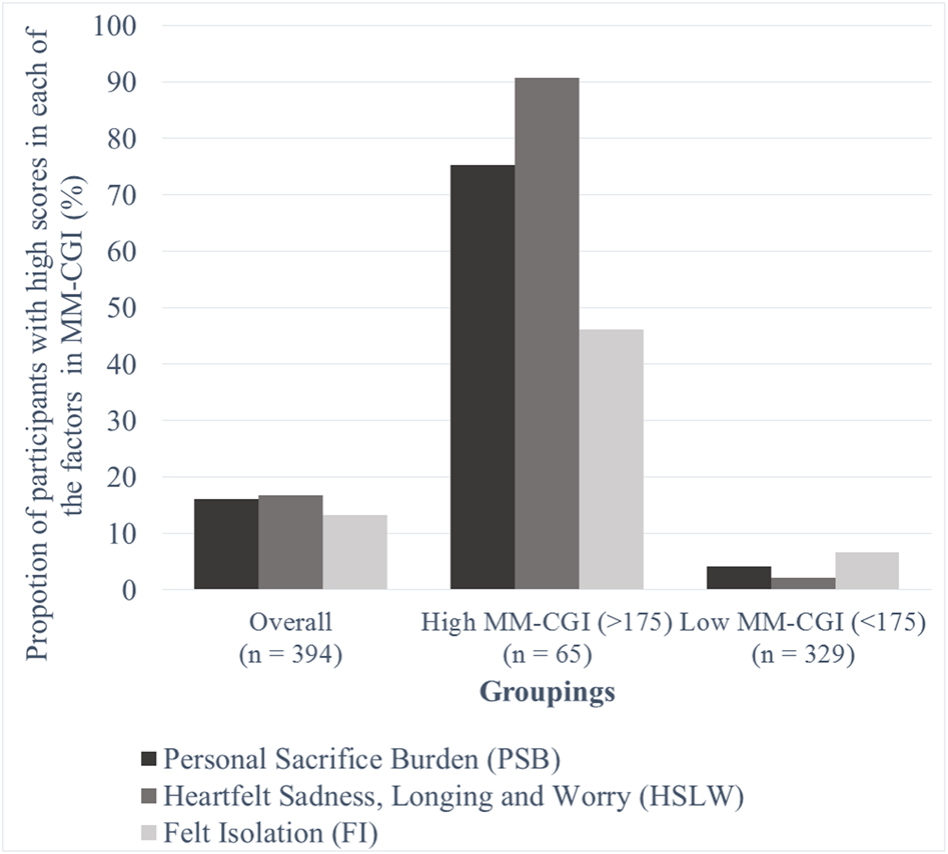
Prevalence of high grief across the three dimensions of loss, stratified by high (>175) or low (<175) total scores on the Marwit‐Meuser Caregiver Grief Inventory [Colour figure can be viewed at http://wileyonlinelibrary.com]

To evaluate the utility of the three dimensions, we further examined the proportion of participants with high subscale scores (above mean + 1 SD) in each of the dimensions. As presented in Figure 3, overall, 16.0% of participants had high scores in *PSB*, 16.8% had high *HSLW*, and 13.2% had high *FI*. Caregivers with high total scores (MM‐CGI > 175) tended to have most difficulty with *HSLW* (90.8%), followed by *PSB* (75.4%) and *FI* (46.2%). Even among caregivers without high total scores (MM‐CGI ≤ 175), some were observed to experience difficulties in *PSB* (4.3%), *HSLW* (2.1%), and *FI* (6.7%).

**Figure 4:**
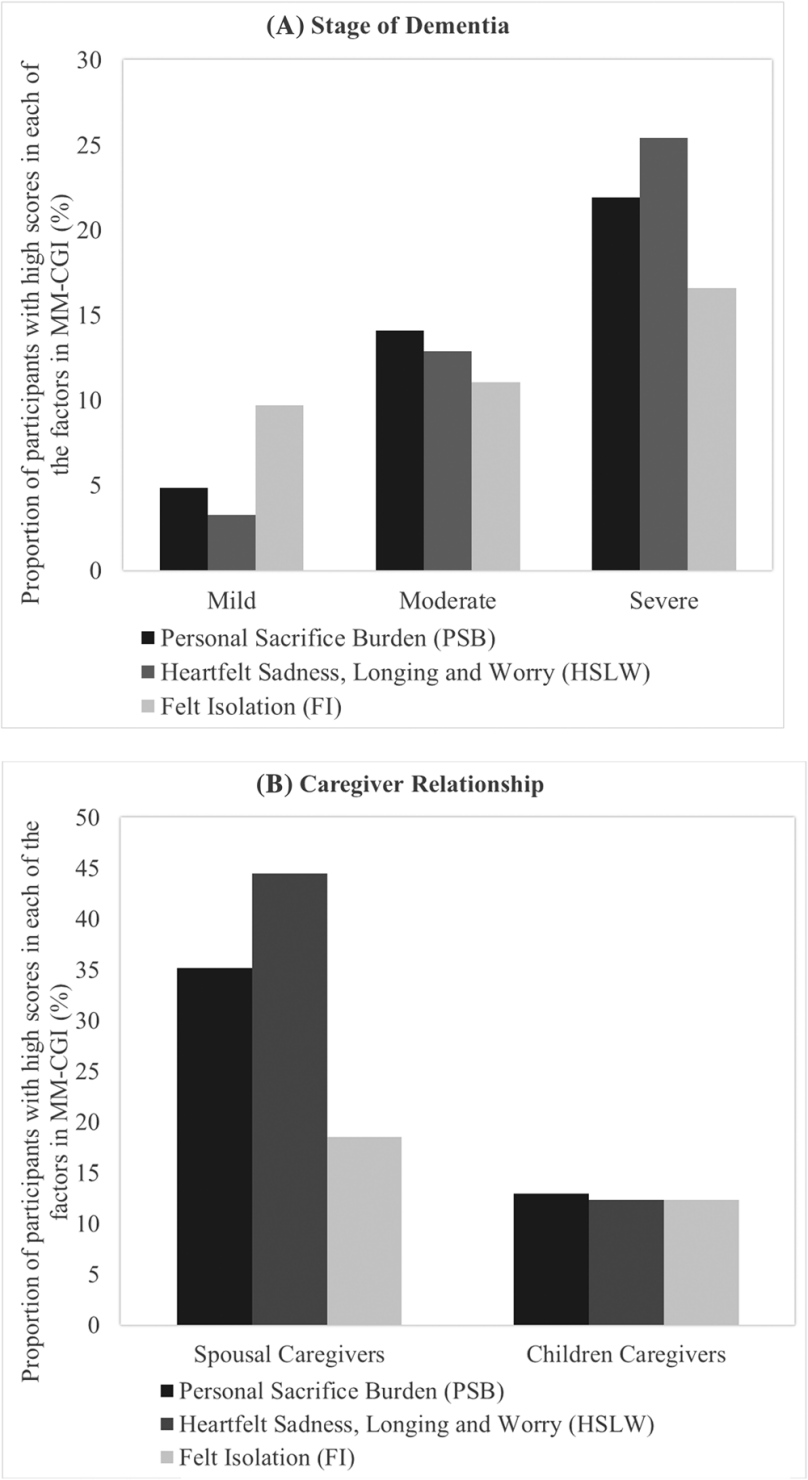
Prevalence of high grief across A, stage of dementia and B, caregiver relationship

We also compared the prevalence of high subscale scores across the stages of dementia and caregiving relationship. The prevalence across the stages of dementia is presented in Figure 4A. In the mild stage, caregivers tended to experience more difficulty with *FI*. In the moderate stage, the primary difficulty was with *PSB*, while in the severe stage, the primary difficulty was related to *HSLW*. The prevalence of high subscale scores across caregiving relationship is presented in Figure 4B. Spousal caregivers experienced the most difficulty in *HSLW* (44.4%), followed by *PSB* (35.2%) and *FI* (18.5%). Notably, children caregivers experienced similar levels of difficulty across *HSLW* (12.4%), *PSB* (12.9%), and *FI* (12.4%).

## 4 DISCUSSION

### 4.1 Summary of findings

This study has evidenced the validity and reliability of the three revised MM‐CGI subscales in capturing the different dimensions of caregiver grief in dementia caregiving. Specifically, the results demonstrated that the subscales possessed adequate internal consistency, test‐retest reliability, construct validity, and known‐group validity. Notably, *FI* exhibited a relatively lower internal consistency among the three dimensions, although it was still above the minimally acceptable reliability indices of ≥0.70. Our results also demonstrated the added utility of the subscales in providing more granular information about the specific aspects of grief experienced by each caregiver. For example, out of the three dimensions of grief, the majority of caregivers who had high total grief scores tended to have high subscores in *HSLW*. Even among caregivers who did not have high total grief scores, some were still observed to exhibit high subscores and were at high risk of having difficulties in specific domains. Moreover, the three dimensions of grief were also expressed differently across the stages of dementia and caregiving relationship. Caregivers were more likely to have difficulties related to *FI* in the mild stage, *PSB* in the moderate stage, and *HSLW* in the severe stage. Comparing across caregiving relationship, spousal caregivers were more likely to experience difficulty in *HSLW*, whereas children caregivers experienced difficulty in all three domains equally.

### 4.2 Interpretation of results

Out of the three factors, *FI* fared poorer in terms of internal consistency reliability. This finding was consistent with a recent study on the MM‐CGI‐SF in a Chinese sample, which reported a lower internal consistency coefficient of 0.63 for FI whereas the other two subscales scored above 0.70.^45^ In contrast, the original validation study in the United States demonstrated internal consistency coefficients of.90 and above for all three subscales.^22^ This may possibly be related to the difficulty of caregivers in our population in consistently identifying with the meaning of the items in the *FI* construct, which encompasses the loss of support. Two types of perceived support have been well‐described in the literature, namely, emotional support and problem‐based support. Emotional support involves reassuring others of one's care, whereas problem‐based support involves providing tangible help to others. Asians have been found to place unequal values on the two types of support, whereas Americans were noted to value both types of support equally.^46^, ^47^ The lack of specification in the type of support referred to in the MM‐CGI could possibly have led to inconsistent interpretations of the items in FI by Singaporean caregivers. In contrast, this would not have been a problem if both types of support were valued equally as found in the American sample, possibly accounting for the higher internal consistency reliability of FI in the original study from the United States.

The poorer internal consistency of FI may also be related to how Cronbach α was computed. Cronbach α is a function of the number of items in a scale and the average inter‐item correlation. As such, the value of Cronbach α becomes smaller with decreasing number of items in the scale.^40^ Evidently, *FI* only had six items, compared with 17 items in *PSB* and 27 items in *HSLW*. Nonetheless, the internal consistency of *FI* in the current study (Cronbach α = .78) is still adequate for psychometric scales and research purposes, although not suitable for individual clinical assessment.^40^

The results on construct validity were consistent with a validation study of the Chinese MM‐CGI‐SF such that out of the three factors, PSB correlated most strongly with caregiver strain, which was similar to the concept of caregiver burden (measured using ZBI) in our study. However, contrary to our results of HSLW correlating most strongly with depression (measured using CES‐D), Chan et al^48^ reported a weaker correlation (*r* = 0.45, *P* < .01) between HSLW and the Chinese CES‐D. Nevertheless, the relationship remained significant and the value was modestly close to our defined strong correlation coefficient threshold of 0.5. Hence, the relationships reported can be construed to be consistent with the assumption that caregivers at risk of high grief may also experience other negative conditions such as caregiver burden or depression. In terms of known‐group validity, Chan et al^48^ also presented corresponding findings of spousal caregivers experiencing a higher level of grief than nonspousal caregivers. In addition, although no significant relationship was found between the stage of dementia and the Chinese MM‐CGI‐SF, Chan et al also similarly noted an increasing trend in the grief level of caregivers according to dementia severity.^48^

Across the stages of dementia, caregivers were more likely to have difficulties related to *FI* in the mild stage, *PSB* in the moderate stage, and *HSLW* in the severe stage. Caregivers may be prone to experiencing more *FI* in the mild stage because family members may underestimate the amount of support a main caregiver requires and may think the illness does not warrant more assistance from them. Furthermore, the main caregiver may not yet be part of a community of other dementia caregivers and may only receive such community support later in the caregiving journey. Caregivers may be at risk of experiencing *PSB* in the moderate stage because PWDs tend to exhibit more behavioral problems during this period, which can adversely affect the caregiver's life.^49^, ^50^ The presence of behavioral problems may lead to a decrease in the caregiver's freedom, causing the caregiver to focus on what one has had to give up (eg, freedom of time) in order to care for the PWD. Caregivers may be at risk of experiencing *HSLW* in the severe stage as the focus of the caregiver shifts from individual losses to PWD‐related losses. At this stage, the identity of the PWD is threatened by dementia as PWDs have a diminished ability to recognize even familiar family members and lose their language capacity, further highlighting the loss of the PWD,^51^ resulting in the caregiver's emotional reactions to such losses.

Our results corresponded to findings of the original MM‐CGI validation paper of PSB showing the highest mean score in the moderate stage of dementia and the original HSL factor (without the worry component) expressing one of the highest mean scores (M = 53.39) in the severe stage (after PSB: M = 57.07).^22^ Similarly, a study examining the MM‐CGI‐SF in a sample of Chinese caregivers also found *PSB* to have the highest mean score in the moderate stage and *HSL* highest in the severe stage.^45^ However, in contrast to our results of *FI* obtaining the highest score in the mild stage of dementia, both studies reported *WFI* to have the lowest mean scores in the mild stage.^22^, ^45^ One possible reason could be the difference in proportion of spousal and child caregivers. Majority of our caregivers were child caregivers (86.3%) whereas the proportion of spousal and child caregivers was more evenly distributed in both Marwit and Meuser's (45 child caregivers and 42 spousal caregivers)^22^ and Li et al's (38 child caregivers and 37 spousal caregivers)^45^ studies. Evidence has suggested that the emotional burdens of *PSB* and *HSL* may be greater for spousal caregivers,^22^, ^48^ leading to higher scores in the two factors compared with *FI*. This is also supported by our results of spousal caregivers to have higher risk in *HSLW* and *PSB* than *FI*, whereas child caregivers experienced similar levels of risk across the three domains. Nonetheless, caution should be exercised when comparing our results to that of other studies. In comparison to the original *WFI* factor, our revised factor, *FI*, excluded the “Worry” component, which was loaded onto *HSLW*. In addition, our data specifically looked at caregivers with high levels of subscores (ie, mean subscale score + 1 SD), whereas the studies by Marwit and Meuser^22^ and Li et al^45^ primarily utilized the mean scores of each factor.

### 4.3 Clinical implications

Overall, the examination of the individual domains of caregiver grief can be relevant to clinical practice in dementia care in the future. First, by examining the individual domains, our results shed light on a specific group of caregivers who are at high risk of experiencing difficulties in specific domains of caregiver grief while their overall grief remained below the cutoff. Although the clinical validity of the total scores and subscores have yet to be validated, our results can lend some credence to the need to explore specific subscales of caregiver grief to identify individuals who may benefit from intervention for those particular dimensions. Second, the granular information provided by the subscales, if shown to be associated with clinical validity in future studies, may benefit care providers in conducting individualized interventions for caregiver grief. Targeting specific domains of caregiver grief in which caregivers may be experiencing more difficulty could increase the effectiveness of interventions. For instance, in terms of dementia severity, interventions intended for caregivers caring for PWDs in the mild stage of dementia should be more attentive towards caregivers experiencing *FI*, whereas interventions for caregivers dealing with severe stage of dementia should be mindful of *HSLW*. Similarly, interventions for spousal caregivers can be more attentive towards *HSLW* and *PSB*. As the original authors believed, emphasis of the three domains is imperative in determining intervention approach.^52^ As for examples of possible interventions for each domain, care providers may consider recommending day care centers, caregiver training programs, and helper training programs for *PSB*, professional grief counseling for *HSLW*, and caregiver support groups for *FI*. In essence, by conducting the initial validation of the individual domains of caregiver grief and identifying at‐risk groups, we hope our results would help pave the way towards future clinical validation of these domains against a clinical definition of grief and help inform individualized caregiver grief assessment and intervention.

We envisage that the utility of the 50‐item MM‐CGI may be more limited in instances where time is of the essence, and only a brief screening is required or where a measure of caregiver grief is only a subset of a larger set of assessments. Shorter screening tools, such as the 18‐item MM‐CGI‐SF,^17^ six‐item MM‐CGI‐Brief Form,^18^ or four‐item Screening Tool for Caregiver Grief^19^ may be more viable under these circumstances. Nevertheless, the full MM‐CGI still remains more sensitive to caregiver grief than the short forms. This is evidenced by the slightly higher internal consistency and test‐retest reliability alphas of MM‐CGI compared to the MM‐CGI‐SF (.90 vs.80 range).^17^, ^21^

### 4.4 Limitations

We acknowledge certain limitations to this study. First, the questionnaires were self‐administered; hence, caregivers with lower literacy may have been under‐represented in our study. Second, we recruited our participants from tertiary care settings; thus, they may not be fully representative of the community. Nonetheless, this may pose less of a problem in the local context as majority of the PWDs in Singapore receive dementia care from tertiary settings, and the two recruitment centers in this study are the only two dementia services that serve the population in the North‐East of Singapore. Third, stage of dementia was assessed through caregiver self‐reports, which may lack the reliability of other staging instruments and objective assessments by health care professionals. Fourth, although we came up with possible explanations for our results, such as the reasons behind each domain obtaining highest scores in different stages of dementia, there may be several other reasons that could explain the same findings. Thus, our interpretations will require further qualitative inquiry in future studies. Fifth, the MM‐CGI — as well as other available caregiver grief scales in the literature — has not been validated against a reference standard based on the clinical definition of grief, which is a potential area for future research. As such, high scores in MM‐CGI or its subdomains do not necessarily imply clinical grief — they are primarily useful to indicate those at a higher risk of caregiver grief (due to the higher than average scores), prompting the need for further clinical evaluations for grief‐related difficulties.

### 4.5 Conclusion

The three subscales of MM–CGI showed adequate psychometric properties, and demonstrated added utility in identifying the specific domains of grief in which caregivers experienced high subscores in (beyond that of overall MM‐CGI score). These three subscales may potentially be useful to care providers in identifying and supporting the emotional needs of caregivers, in a more focused and individualized manner. They help to pave the way for individualized caregiver grief assessments and intervention in both clinical and research settings.

## CONFLICTS OF INTEREST

None declared.

## FUNDING

This research was supported by the Singapore Ministry of Health's National Medical Research Council under the Centre Grant Program (Grant No.: NMRC/CG/004/2013). It also received pilot funding from the National University of Singapore. Separately, the corresponding author (T.M.L.) was supported by research grants under the Singapore Ministry of Health's National Medical Research Council (Grant No.: NMRC/Fellowship/0030/2016 and NMRC/CSSSP/0014/2017). The funding sources had no involvement in any part of the project.

## DATA SHARING AND DATA ACCESSIBILITY

The data that support the findings of this study are available from the corresponding author upon reasonable request.

## Supporting information

Table S1. Internal‐consistency reliability of the factors of MM‐CGI
