# Supplementary material for: The three dimensions of caregiver grief in dementia caregiving: Validity and utility of the subscales of the Marwit‐Meuser Caregiver Grief Inventory
Source: Int J Geriatr Psychiatry. 2019 Dec 6;35(2):213–22. doi: 10.1002/gps.5238 (PMC7004032; doi:10.1002/gps.5238)
Supplement: Supplementary file 1 — Table S1. Internal‐consistency reliability of the factors of MM‐CGI [file GPS-35-213-s001.docx]

**SUPPLEMENTARY MATERIAL**

**Supplementary Material 1.** Internal-consistency reliability of the factors of MM-CGI

| MM-CGI items within the revised factors | Item-rest correlation ^a^ | Cronbach’s α if item deleted ^b^ |
| --- | --- | --- |
| ***Factor 1: Personal sacrifice burden (Cronbach’s α=0.95)*** |  |  |
| 1. I’ve had to give up a great deal to be a caregiver^c^. | 0.69 | 0.94 |
| 3. I feel I am losing my freedom^c^. | 0.80 | 0.94 |
| 4. My physical health has declined from the stress of being a caregiver. | 0.72 | 0.94 |
| 7. I carry a lot of stress as a caregiver. | 0.75 | 0.94 |
| 11. My personal life has changed a great deal. | 0.78 | 0.94 |
| 17. I feel this constant sense of responsibility and it just never leaves. | 0.69 | 0.94 |
| 20. I can’t feel free in this situation. | 0.79 | 0.94 |
| 25. I feel so frustrated that I often ignore him/her. | 0.47 | 0.95 |
| 28. This is requiring more emotional energy and determination than I ever expected. | 0.72 | 0.94 |
| 29. I will be tied up with this for who knows how long^c^. | 0.68 | 0.95 |
| 35. His/her death will bring me renewed personal freedom to live my life. | 0.46 | 0.95 |
| 39. Independence is what I’ve lost…I don’t have the freedom to go and do what I want^c^. | 0.81 | 0.94 |
| 40. I’ve had to make some drastic changes in my life as a result of becoming a caregiver. | 0.79 | 0.94 |
| 41. I wish I had an hour or two to myself each day to pursue personal interests^c^. | 0.67 | 0.95 |
| 42. I’m stuck in this caregiving world and there’s nothing I can do about it^c^. | 0.77 | 0.94 |
| 44. What upset me most are the things I have to give up. | 0.73 | 0.94 |
| 2. I miss so many of the activities we used to share. | 0.56 | 0.95 |
|  |  |  |
| ***Factor 2: Worry, heartfelt sadness and longing (Cronbach’s α=0.96)*** |  |  |
| 9. I have this empty, sick feeling knowing that my loved one is “gone” ^c^. | 0.60 | 0.96 |
| 14. I feel terrific sadness. | 0.81 | 0.96 |
| 15. This situation is totally unacceptable in my heart. | 0.76 | 0.96 |
| 18. I long for what was, what we had and shared in the past^c^. | 0.61 | 0.96 |
| 19. I could deal with other serious disabilities better than with this^c^. | 0.46 | 0.96 |
| 27. I’m angry at the disease for robbing me of so much. | 0.69 | 0.96 |
| 30. It hurts to put her/him to bed at night and realize that she/he is “gone” ^c^. | 0.63 | 0.96 |
| 31. I feel very sad about what this disease has done^c^. | 0.66 | 0.96 |
| 36. I feel powerless. | 0.65 | 0.96 |
| 37. It’s frightening because you know doctors can’t cure this disease, so things only get worse. | 0.66 | 0.96 |
| 38. I’ve lost other people close to me, but the losses I’m experiencing now are much more troubling^c^. | 0.72 | 0.96 |
| 43. I can’t contain my sadness about all that’s happening. | 0.81 | 0.96 |
| 48. I’ve had a hard time accepting what is happening. | 0.75 | 0.96 |
| 50. I wish this was all a dream and I could wake up back in my old life. | 0.63 | 0.96 |
| 6. I don’t know what is happening. I feel confused and unsure. | 0.65 | 0.96 |
| 10. I feel anxious and scared. | 0.72 | 0.96 |
| 12. I spend a lot of time worrying about the bad things to come^c^. | 0.75 | 0.96 |
| 13. Dementia is like a double loss…I’ve lost the closeness with my loved one and connectedness with my family^c^. | 0.73 | 0.96 |
| 16. My friends simply don’t understand what I’m going through^c^. | 0.52 | 0.96 |
| 22. I’m at peace with myself and my situation in life ^d^. | 0.47 | 0.96 |
| 23. It’s a life phase and I know we’ll get through it ^d^. | 0.35 | 0.96 |
| 26. I am always worrying. | 0.75 | 0.96 |
| 32. I feel severe depression. | 0.77 | 0.96 |
| 33. I lay awake most nights worrying about what’s happening and how I’ll manage tomorrow^c^. | 0.78 | 0.96 |
| 46. I think I’m denying the full impact of this disease for my life. | 0.59 | 0.96 |
| 21. I’m having trouble sleeping. | 0.66 | 0.96 |
| 49. The demands on me are growing faster than I ever expected. | 0.75 | 0.96 |
|  |  |  |
| ***Factor 3: Felt isolation*** ***(Cronbach’s α=0.78)*** |  |  |
| 5. I have nobody to communicate with^c^. | 0.56 | 0.74 |
| 8. I receive enough emotional support from others ^d^. | 0.44 | 0.77 |
| 24. My extended family has no idea what I go through in caring for him/her. | 0.60 | 0.73 |
| 34. The people closest to me do not understand what I’m going through^c^. | 0.64 | 0.72 |
| 45. I’m managing pretty well overall ^d^. | 0.35 | 0.79 |
| 47. I get excellent support from members of my family ^d^. | 0.58 | 0.74 |

*Notes:* MM-CGI = Marwit-Meuser Caregiver Grief Inventory.

^a^ Correlation between the item and the summated score for all other items from the same factor.

^b^ Cronbach’s α of the respective subscale if the item was deleted from the subscale.

^c^ Items used in MM-CGI-SF.

^d^ Items in reverse wordings.
